# SABRE hyperpolarized anticancer agents for use in ^1^H MRI

**DOI:** 10.1002/mrm.29166

**Published:** 2022-03-07

**Authors:** Elizabeth J. Fear, Aneurin J. Kennerley, Peter J. Rayner, Philip Norcott, Soumya S. Roy, Simon B. Duckett

**Affiliations:** ^1^ Hull York Medical School University of York York United Kingdom; ^2^ Centre for Hyperpolarisation in Magnetic Resonance (CHyM) University of York York United Kingdom; ^3^ Research School of Chemistry Australian National University Canberra Australia; ^4^ School of Chemistry University of Southampton Southampton United Kingdom; ^5^ Defence Science and Technology Laboratory (DSTL) Salisbury United Kingdom

**Keywords:** cancer, hyperpolarization, MRS(I), SABRE, thienopyridazines

## Abstract

**Purpose:**

Enabling drug tracking (distribution/specific pathways) with magnetic resonance spectroscopy requires manipulation (via hyperpolarization) of spin state populations and targets with sufficiently long magnetic lifetimes to give the largest possible window of observation. Here, we demonstrate how the proton resonances of a group of thienopyridazines (with known anticancer properties), can be amplified using the para‐hydrogen (*p*‐H_2_) based signal amplification by reversible exchange (SABRE) hyperpolarization technique.

**Methods:**

Thienopyridazine isomers, including a ^2^H version, were synthesized in house. Iridium‐based catalysts dissolved in a methanol‐*d*_4_ solvent facilitated polarization transfer from *p*‐H_2_ gas to the target thienopyridazines. Subsequent SABRE ^1^H responses of hyperpolarized thienopyridazines were completed (400 MHz NMR). Pseudo‐singlet state approaches were deployed to extend magnetic state lifetimes. Proof of principle spectral‐spatial images were acquired across a range of field strengths (7T‐9.4T MRI).

**Results:**

^1^H‐NMR signal enhancements of −10,130‐fold at 9.4T (~33% polarization) were achieved on thieno[2,3‐*d*]pyridazine (T[2,3‐*d*]P), using SABRE under optimal mixing/field transfer conditions. ^1^H T_1_ lifetimes for the thienopyridazines were ~18‐50 s. Long‐lived state approaches extended the magnetic lifetime of target proton sites in T[2,3‐*d*]P from an average of 25‐40 seconds. Enhanced in vitro imaging (spatial and chemical shift based) of target T[2,3‐*d*]P was demonstrated.

**Conclusion:**

Here, we demonstrate the power of SABRE to deliver a fast and cost‐effective route to hyperpolarization of important chemical motifs of anticancer agents. The SABRE approach outlined here lays the foundations for realizing continuous flow, hyperpolarized tracking of drug delivery/pathways.

## 1 INTRODUCTION

Magnetic resonance (MR) remains a key non‐invasive technology for the diagnosis of disease by tracing aberrant cellular function.^1^, ^2^ Although MR provides both fundamental molecular data (spectra) alongside macroscopic structural information (images), inherent sensitivity is limited by low thermal polarization. Strong magnetic fields (>7T), high concentrations, and signal averaging can overcome this limitation. However, such solutions can be impractical for *in vivo* metabolomics or drug tracking.

Cutting edge hyperpolarization technologies,^3^, ^4^, ^5^, ^6^, ^7^, ^8^ offering non‐destructive manipulation of quantum spin state populations present as a promising alternative. Success relies on the achievement of remarkable improvements (×10 000) in signal intensity compared to thermal based MR practices.^6^ Spin exchange optical pumping (SEOP) techniques (delivering hyperpolarized ^3^He^9^ and ^129^Xe^10^, ^11^) are now permitting exploration of alveolar geometry, complementary gas flow, and diffusion MR measurements in the lungs.^9^, ^10^, ^11^, ^12^, ^13^, ^14^ In the field of biomedicine, dissolution dynamic nuclear polarization (d‐DNP) is emerging as a leading methodology for real‐time *in vivo* metabolomics in oncology and cardiology.^15^, ^16^, ^17^, ^18^

Although next generation d‐DNP polarizers using cryo‐cooling^19^, ^20^ or multi‐sample chambers^20^ are under development, research into complementary hyperpolarization technologies offering continuous delivery of polarized agents is needed. One method, para‐hydrogen induced polarization (PHIP), uses para*‐*hydrogen (*p*‐H_2_) as the source of polarization, which has a long magnetic lifetime and can be readily prepared in large volumes.^21^, ^22^ The dormant magnetization of (*p*‐H_2_) can be quickly released through chemical transfer into a dehydrogenated target material. PHIP‐side arm hydrogenation (SAH) expands on this route by releasing the final molecular probe via hydrolysis.^23^ *In vivo* metabolic images using PHIP‐SAH hyperpolarized [1‐^13^C]pyruvate permit monitoring of altered cardiac metabolism.^7^, ^24^, ^25^

**FIGURE 1:**
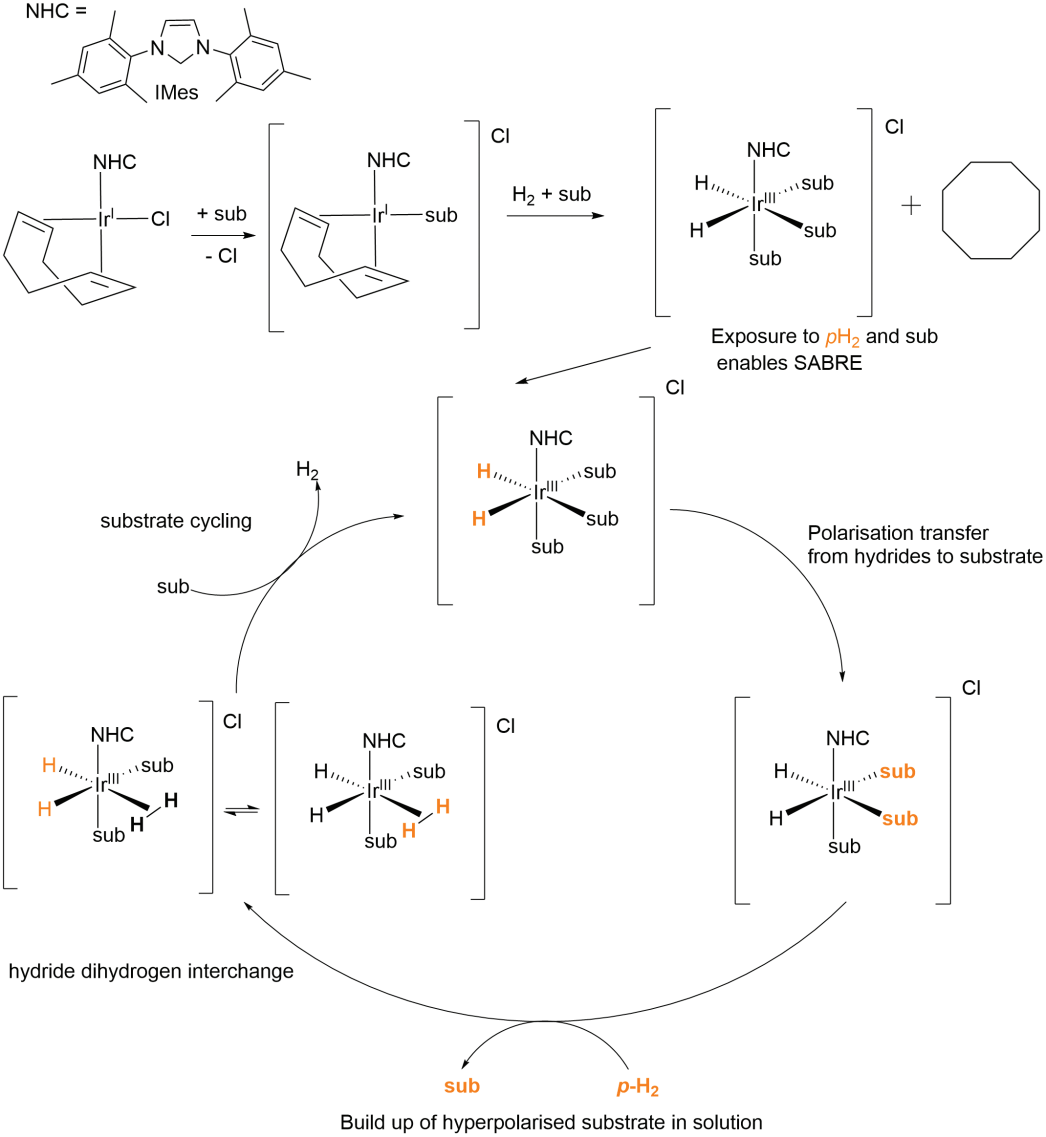
Formation of a SABRE active catalyst proceeds alongside the hydrogenation of cyclooctadiene (COD). The cycle shows ligand exchange and the transfer of polarization from the *p*‐H_2_ derived hydride ligands to the substrate

*p*‐H_2_ induced polarization can also be implemented without changing the chemical identity of the agent (substrate) of interest. This is achieved via signal amplification by reversible exchange (SABRE)^3^ where a metal framework temporarily, and reversibly, binds both *p*‐H_2_ and the target substrate (Figure 1). When *p*‐H_2_ binds to form metal hydrides the symmetry of its singlet state is broken and polarization is delivered to the target substrate instantaneously via scalar coupling. Because bound ligands are in continuous exchange with those in solution, hyperpolarized agent builds up over a matter of seconds (Figure 1). A suitable precatalyst for this process is [IrCl(COD)(IMes)], selected because of its ready synthesis and stability, however, it is possible to tailor the outcome of SABRE for specific substrates by changing the steric and electronic properties of the *N*‐heterocyclic carbene (NHC) ligand.^26^ This has enabled ^1^H NMR signals associated with up to 60% magnetic state purity to be recorded.^27^ Importantly, what sets SABRE apart, is the ability to repeatedly or continuously create hyperpolarized solutions in seconds allowing continuous delivery of hyperpolarized agents.^28^ Aligned with the wide range of possible target substrates, one can envisage the use of SABRE for tracking/imaging drug delivery.

**FIGURE 2:**
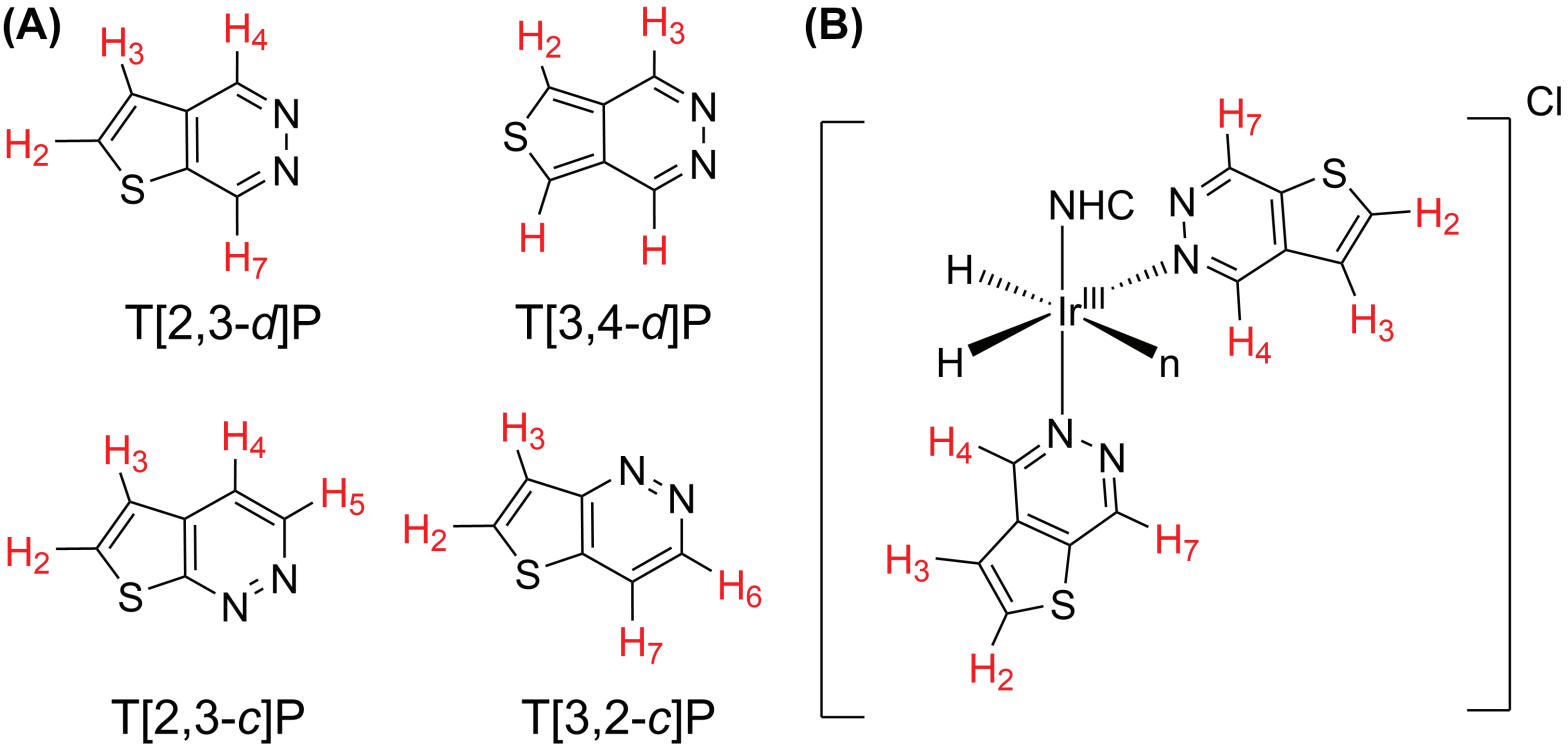
(A) Structure of the thienopyridazines synthesised^39^, ^40^ and hyperpolarized in this study by SABRE, Thieno[2,3‐*d*]pyridazine, (T[2,3‐*d*]P), Thieno[3,4‐*d*]pyridazine (T[3,4‐*d*]P), Thieno[2,3‐*c*]pyridazine (T[2,3‐*c*]P), Thieno[3,2‐*c*]pyridazine (T[3,2‐*c*]P). (B) Potential active SABRE catalyst [IrH_2_(IMes)(T[2,3‐*d*]P)_3_]Cl; n is T[2,3‐*d*]P and omitted for clarity

The present study investigates ^1^H SABRE hyperpolarization efficiency (via ^1^H NMR signal enhancements and T_1_ relaxation times) for a family of synthesized thienopyridazine (TP) isomers (Figure 2A). Pyridazine chemistry is a major research interest because its derivatives exhibit a wide variety of biological roles that include anti‐inflammatory,^29^, ^30^ anticancer,^31^, ^32^ antiviral,^33^ and antimicrobial^34^ activity. Specifically, the TP motif is found in drugs that demonstrate anticancer activity (e.g., T[2,3‐*d*]P is found in IκB kinase [IKKs] inhibitors.^35^, ^36^, ^37^). This class of compounds also possesses an N‐heterocyclic ring, which makes them suitable as ligands for coordination to a SABRE catalyst^3^, ^27^, ^38^ (Figure 2B). We synthesised^39^, ^40^ and tested the four TP isomers, alongside studies involving the inclusion of a fully deuterated isotopologue as co‐ligand to increase percentage polarization. To extend magnetization lifetimes and enhance the timing window for subsequent imaging we also explored application of pseudo‐long lived state strategies.^41^, ^42^ Following optimization, the imaging capability of these hyperpolarized drug motifs was tested *in vitro*. Together this data shows recent development work toward potential drug tracking for biomedical ^1^H MR imaging.

## 2 METHODS

Unless otherwise specified SABRE polarization transfer experiments used 5 mm NMR tubes fitted with a J. Young valve that contained 4.5 mM solutions of [IrCl(COD)(IMes)] in methanol‐*d*_4_ where the target TP substrate was present in a 1:4 ratio of catalyst to substrate. Samples were degassed by freeze‐pump‐thaw cycles to remove dissolved oxygen before precatalyst activation and SABRE (Figure 1).

Samples were exposed to 3 bar(g) *p*H_2_ gas and mixed to start the polarization exchange either (1) manually (simply through vigorous shaking) for 10 s (method 1); or (2) via an automated polarizer (method 2). Polarization transfer was implemented at ~60 G for ^1^H in the fringe field of the NMR spectrometer^43^, ^44^, ^45^ or in a hand‐held magnet array.^46^

MR signal detection was completed using a BBI probe on 400 MHz or 500 MHz spectrometers (Bruker, Ettlingen, Germany). Polarization levels were assessed using a pulse acquire sequence (90° flip angle), with a receiver gain of 1.

In method 1, the source of *p‐*H_2_ was a custom‐made *p*‐H_2_ rig.^47^ The source of *p‐*H_2_ for method 2 was a FDGS‐Alliance (Evry, France) hydrogen generator linked to a Bruker *p*‐H_2_ generator.

Method 2 allowed for efficient delivery of the sample into the detector following *p‐*H_2_ addition (nitrogen gas shuttles solutions from the solenoid where SABRE takes place to the NMR detector).^48^ A microcontroller sets the desired parameters (e.g., *p*‐H_2_ bubbling time, mixing field, time to transfer, etc.) and timing, which is controlled within the pulse sequence.^49^

Six catalyst precursors were tested with T[2,3‐*d*]P. [IrCl(COD)(IMes)],^50^ and [IrCl(COD)(1,3‐bis(2,3,6‐trimethylphenyl)‐4,5‐dihydroimidazol‐2‐ylidene)], [IrCl(COD)](SIMes)^50^; the deuterated versions of both, [IrCl(COD)(*d*_22_‐1,3‐bis(2,4,6‐trimethyl)‐imidazol‐2‐ylidene)] ([IrCl(COD)(*d*_22_‐IMes)])^51^ and [IrCl(COD)(*d*_22_‐1,3‐bis(2,3,6‐trimethylphenyl)‐4,5‐dihydroimidazol‐2‐ylidene)] [IrCl(COD)(*d*_22_‐SIMes)]^27^; [IrCl(COD)(1,3‐bis(4‐chloro‐2,6‐dimethylphenyl)imidazol‐2‐ylidine)],^27^ [IrCl(COD)(X)] and [IrCl(COD)(1,3‐bis(4‐(dimethylamino)‐2,6‐dimethylphenyl)imidazol‐2‐ylidine)]^27^ [IrCl(COD)(Y)], (Figure 6). The synthesis of the deuterated co‐ligand is detailed in Supporting Information section 1.

### 2.1 Signal enhancement factors calculations and errors and percentage polarization

NMR signals enhancement, ε, for individual resonances were calculated using Equation (1).

(1) $$\varepsilon =\frac{S_{\mathrm{hyp}}}{S_{\mathrm{ref}}}.\frac{N_{\mathrm{ref}}}{N_{\mathrm{hyp}}},$$

where S_hyp_ = signal integral from the hyperpolarised spectrum, S_ref_ = signal integral from unpolarized sample at thermal equilibrium, both acquired across N respective repeats. All NMR experimental parameters, except the number of scans, remained the same for hyperpolarized and thermal measurements. For ^1^H detection N = 1 for both hyperpolarized and reference measures. Standard errors were calculated across n (typically between 5 and 8) repeats.

Enhancement was converted into fractional (percentage) polarization, P using Equation (2).

(2) $$P=\;\frac{\gamma B_{0}\;\hbar }{2\;k_{B}T}\;.\;\varepsilon ,$$

where γ is the gyromagnetic ratio of the nuclei, B_0_ = static magnetic field of measurement, ħ = reduced Planck’s constant, k_B_ = Boltzmann constant and T = temperature at which the measurement takes place.

### 2.2 Signal lifetime measurements

T_1_ was determined by a standard inversion recovery experiment. Integrated data points are fitted to Equation (3).

(3) $$S_{z}\left(\tau \right)=S_{0}\left(1‐2e\left(\frac{‐\tau }{T_{1}}\right)\right),$$

where S_z_(τ) are the signal amplitudes at times τ and S_0_ is a constant. τ is varied between 0 and 5 × T_1_ over a series of experiments. A minimum delay of 5 × T_1_ (estimated) was used to ensure full magnetization relaxation and thereby improve the estimate of T_1_.

Creation of a long‐lived pseudo singlet state (LLS), to extend magnetization relaxation times, requires a molecule containing a pair of spin ½ nuclei (of the same isotopic type, e.g., ^1^H), a difference in chemical shift, Δδ, and a mutual scalar coupling, *J*. The LLS created is not a true singlet/eigenstate (with zero dipole‐dipole relaxation) and as such, without intervention will eventually evolve into states prone to T_1_ relaxation mechanisms. However, if maintained in this pseudo‐singlet state for long enough (through RF in the form of a “spin‐lock”^52^, ^53^or field strength manipulation)^41^, ^54^ the magnetic states decay with a longer time constant, T_LLS_.

**FIGURE 3:**
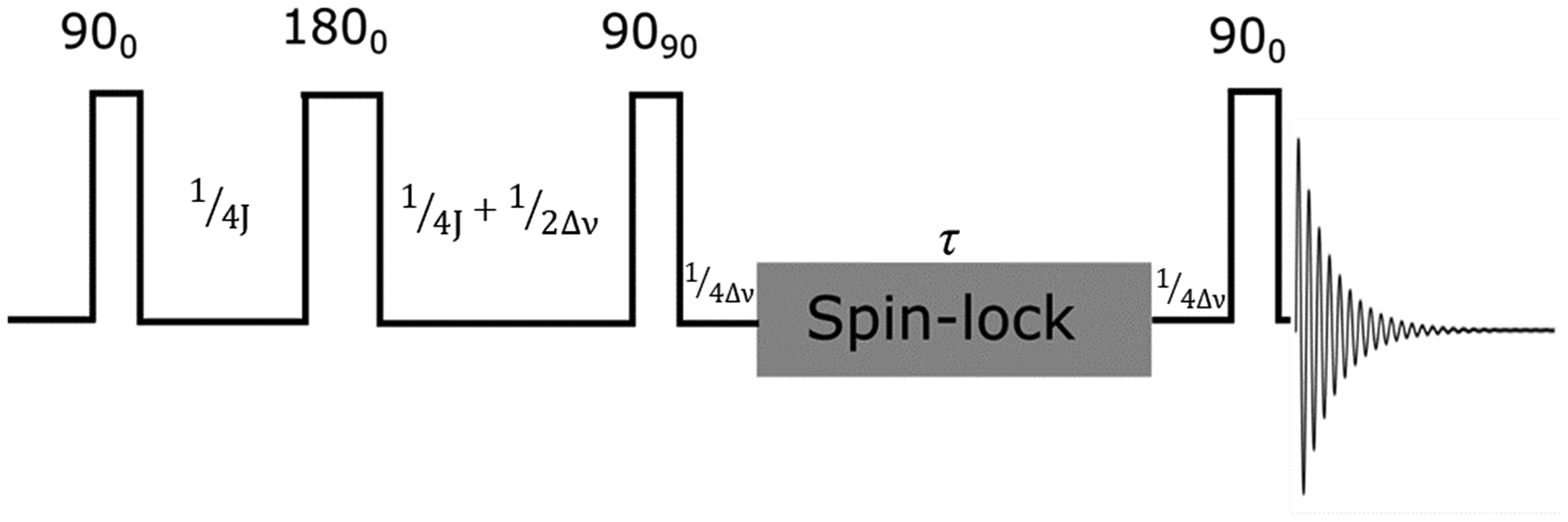
LLS pulse sequence with delays 1/4J, 1/4J + 1/2Δν, 1/4Δν and τ where J and Δν denote the scalar‐coupling constant and chemical shift difference between the associated spin pair in Hertz, respectively. A Waltz‐16 composite pulse of 1 KHz amplitude was used as the spin‐lock (with duration *τ*) in all cases

A spin‐lock long‐lived state pulse sequence^42^ was applied (Figure 3) and the extended decay time constant T_LLS_ estimated. Spin‐lock duration (τ) was varied between 0 and 250 s while keeping all other sequence timing parameters (related to *J* coupling and chemical shift constants) unchanged. Integrated amplitudes S_z_(τ) were fitted to a bi‐exponential function (Equation 4) to explore the efficiency of the spin locking sequence.

(4) $$S_{z}\left(\tau \right)=S_{\mathrm{LSS}}e^{‐\tau \;/\;T_{\mathrm{LLS}}}+S_{T}e^{‐\tau \;/\;T_{T}},$$

where S_LLS_ and S_T_ represent the inherent magnetization in either a slow decaying long‐lived state (LLS) or a normal dipole‐dipole decaying triplet state (T). It is noted that this is a simplified model that assumes no mixing of the two states after spin‐locking.

### 2.3 Imaging

The imaging potential for SABRE hyperpolarized TP was tested using both (1) a 9.4T Bruker vertical bore NMR system equipped with a micro imaging gradient system (1 T/m) and a standard 30 mm inner diameter (ID) ^1^H/^13^C double resonance birdcage for RF transmission and reception; and (2) a 7T preclinical Bruker horizontal bore (BioSpec 70/30) imaging system with a preinstalled BGA‐12S gradient coil (600 mT/m) and an 82 mm ID ^1^H quadrature birdcage resonator.

*In vitro* image acquisition used a single shot rapid acquisition with relaxation enhancement (RARE) imaging sequence (matrix size 128 × 128; FOV 20 × 20 mm; slice thickness 10 mm; centric encoded, RARE factor 128; minimum TE = 20 ms). MRSI acquisition used CSI (image size = 16 × 16; FOV = 35 × 35 mm; data points = 604 band/sweep width = 30 ppm; center frequency = 4.7 ppm; TE = 1.099 ms; TR = 289.021 ms; flip angle = 10°; slice thickness varied from 0.5 mm to 20 mm) or echo planar spectroscopic imaging (EPSI), (image size = 64 × 64; data points = 256; FOV = 35 × 35 mm; band/sweep width = 15.6 ppm; center frequency/O1P = 4.7 ppm; TE = 4 ms; TR = 93.75 ms; flip angle = 15°; slice thickness varied from 0.5 mm to 20 mm). For EPSI experiments, the center frequency of the scanner was set on the water signal at δ 4.7, and automatic shimming, RF power calibration, and N/2 Nyquist ghost correction were completed on a water phantom (10 mm NMR tube filled with 3 ml of water). Peak integrals were calculated in MATLAB (MathWorks, Natick, MA) to generate a signal map, which was overlaid onto appropriate reference ^1^H spin‐echo/gradient echo images.

For imaging, both a 1:4 (optimized ratio for signal enhancement) and 1:20 (greater signal‐to‐noise) ratio of catalyst to substrate‐based samples were tested.

## 3 RESULTS

### 3.1 ^1^H NMR SABRE hyperpolarization experiments

**FIGURE 4:**
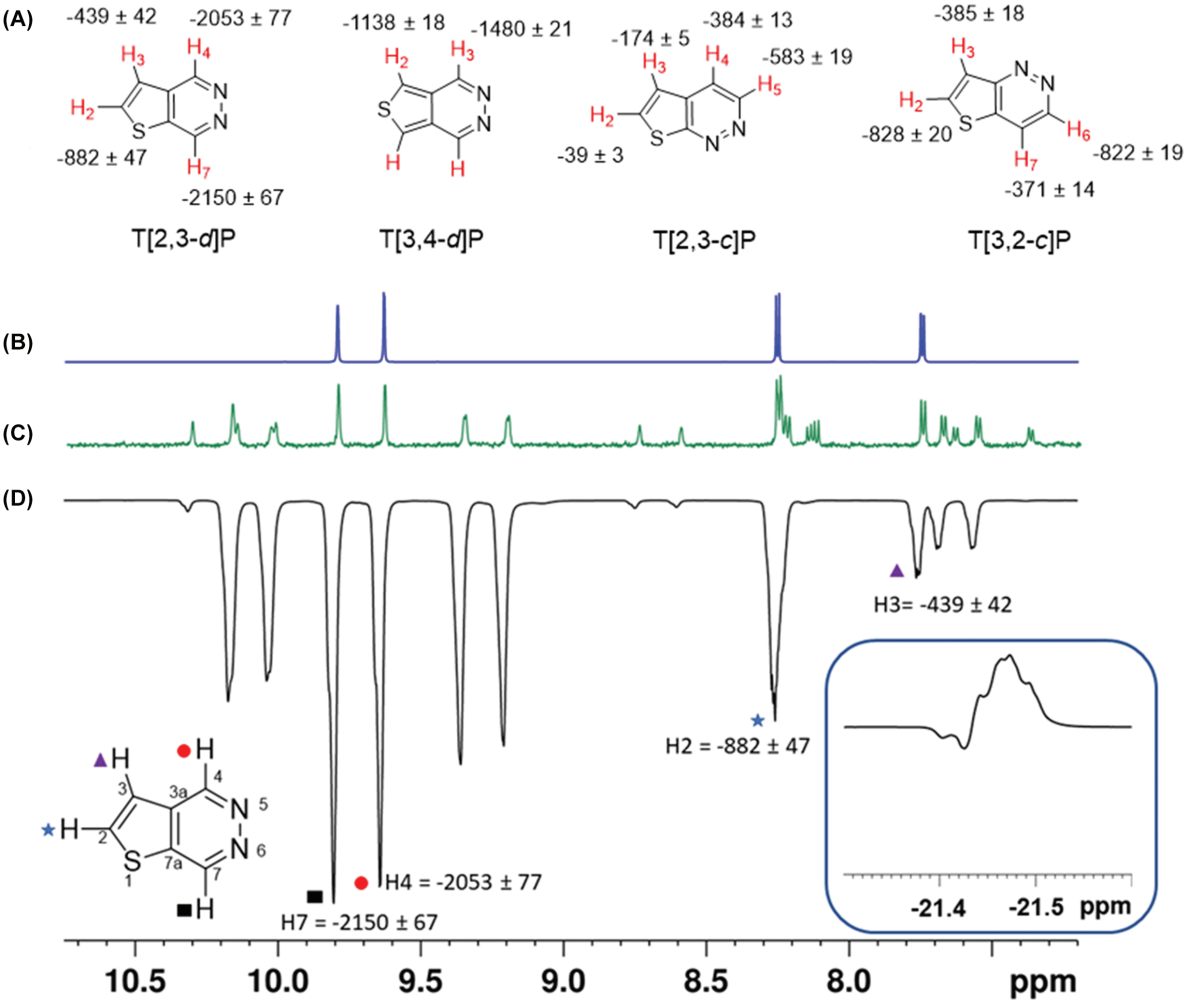
(A) Attributed T[2,3‐*d*]P, T[3,4‐*d*]P, T[2,3‐*c*]P and T[3,2‐*c*]P SABRE enhancement levels resulting from a substrate to [IrCl(COD)(IMes)] ratio of 4 to 1 under 3 bar(g) of *p*‐H_2_. (B) ^1^H NMR control spectrum of T[2,3‐*d*]P at thermal equilibrium. (C) ^1^H NMR spectrum of the reaction mixture for T[2,3‐*d*]P with a vertical expansion of 64 times relative to (D) the result under SABRE conditions. The labeled resonances, indicated with colored symbols, show the free hyperpolarized T[2,3‐*d*]P response. The other unlabeled resonances are in the catalyst, the insert shows the hydride region

A typical SABRE hyperpolarized ^1^H NMR spectrum of T[2,3‐*d*]P achieved using [IrCl(COD)(IMes)] (method 1) is shown in Figure 4 alongside the thermally polarized equivalent, which is vertically magnified 64 times to provide perspective. Bound T[2,3‐*d*]P resonances in [Ir(H)_2_(IMes)(T[2,3‐*d*]P)_3_] and free substrate peaks are observed with enhancement. A signal enhancement of −2150 ± 67 was determined for H7 of the free substrate, (~6.9% ± 0.2% polarization) after SABRE. The highest signal enhancement found for a bound T[2,3‐*d*]P peak was −1576 ± 50, on the signal at 9.3 ppm. Similar results were collected for the remaining TP isomers, with T[2,3‐*d*]P proving to give the greatest signal improvements as summarized in Figure 4A.

### 3.2 Signal lifetime measurements

Inversion recovery‐based T_1_ values were measured for all proton sites in each of the four TP isomers (Table 1). Thermal measurements were made with and without the iridium catalyst. Signal lifetime for the H7 site (expressing the highest SABRE enhancement) in T[2,3‐*d*]P decreased from 49.45 s ± 8.10 s to 7.28 s ± 0.10 s in the presence of the catalyst.

**TABLE 1:** Signal enhancement and relaxation data (T_1_ and T_LLS_) associated with thienopyridazines in the absence and in the presence of [IrCl(COD)(IMes)] in a 1:4 catalyst: substrate ratio using a high field spin‐lock in methanol‐*d*_4_

Substrate	Res.	ε	Substrate alone		Substrate with catalyst	
			T_1_ (s)	T_LLS_ (s)	T_1_ (s)	T_LLS_ (s)
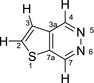	H7	−2150 ± 67	49.45 ± 8.10	–	7.28 ± 0.10	–
	H4	−2053 ± 77	24.88 ± 0.94		5.13 ± 0.06	
	H2	−882 ± 47	27.87 ± 1.50	40.02 ± 2.89	7.50 ± 0.11	9.00 ± 0.43
	H3	−439 ± 42	17.53 ± 0.40		4.64 ± 0.07	
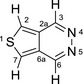	H3	−1480 ± 21	25.86 ± 0.17	–	5.44 ± 0.07	–
	H2	−1138 ± 18	29.10 ± 0.21	–	9.41 ± 0.10	–
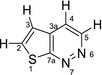	H5	−583 ± 19	21.10 ± 0.49	–	3.19 ± 0.03	–
	H4	−384 ± 13	18.48 ± 0.16		5.21 ± 0.07	
	H2	−174 ± 5	38.08 ± 3.82	32.21 ± 1.64	7.49 ± 0.16	2.34 ± 0.40
	H3	−39 ± 3	28.24 ± 1.04		5.61 ± 0.17	
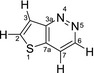	H6	−822 ± 19	18.68 ± 0.18	–	2.90 ± 0.05	–
	H7	−371 ± 14	20.08 ± 0.41		4.96 ± 0.07	
	H2	−828 ± 20	25.80 ± 0.80	21.69 ± 1.51	6.36 ± 0.08	5.20 ± 1.25

LLS preparation using this spin‐locking approach requires neighboring weakly coupled protons exhibiting a Δδ > *J* coupling. It was recognized that the pairs of protons H2/H3 in T[2,3‐*d*]P, H2/H3 and H4/H5 in T[2,3‐*c*]P and H2/H3 and H6/H7 in T[3,2‐*c*]P could be potential sites for the creation of pseudo singlet state for storing the latent magnetization. For example, the *J* coupling value and chemical shift difference of H2/H3 in T[2,3‐*d*]P were 5.3 Hz and 202.5 Hz, respectively.

**FIGURE 5:**
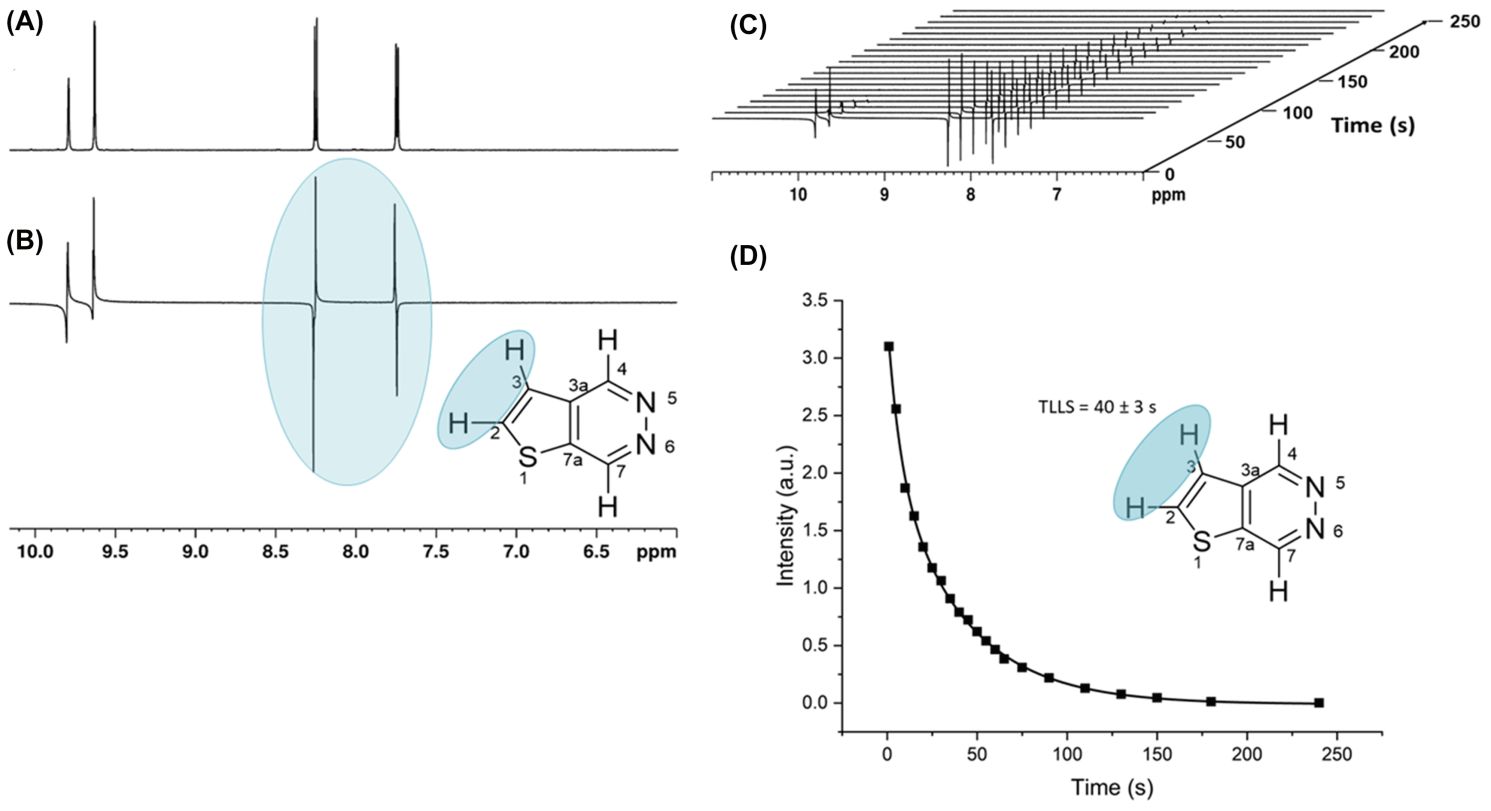
(A) ^1^H NMR spectrum of T[2,3‐*d*]P in methanol‐*d*_4_ showing the 4 proton resonances including the 2 doublets arising from the mutually coupled H2 and H3. (B) Characteristic antiphase peaks observed in the read‐out stage of the LLS experiment of the pseudo‐singlet created between H2 and H3. The highlighted locations indicate both the NMR peaks and the protons within the molecule where the pseudo‐singlet state has been created. (C) Stacked array of NMR spectra resulting from the LLS experiment showing the decay of the long‐lived singlet in the read‐out stage. (D) Exponential decay of the singlet state created between H2 and H3 in T[2,3‐*d*]P using the LLS pulse sequence in high field. The black squares indicate the intensity of the combined NMR signal from the pseudo singlet state in the read‐out stage of the LLS experiment

These values were used to parameterize the pulse timings of the spin‐lock pulse sequence (Figure 3). Figure 5B shows the resultant spin locked spectrum following induction of the pseudo singlet state directly compared to the standard single pulse approach (Figure 5A).

T[3,4‐*d*]P is symmetric and, does not possess neighboring proton spins with a mutual *J* coupling. It was, therefore, not amenable to generation of a pseudo singlet state.

To quantify the time constant of the induced pseudo singlet state twenty spin‐lock experiments were run with increasing storage time (Figure 5C). These time‐resolved spectra clearly show that the resonances at 9.80 and 9.64 ppm (H7 and H4, respectively), which are not involved in the singlet state, relax rapidly. In contrast, the H2 and H3 resonances at 8.23 and 7.75 ppm have a much longer lifetime (a key indicator that pseudo singlet state storage of magnetization is achieved).

Summed integral areas for both H2 and H3 resonances were fitted to Equation (4) assuming the existence of fast decaying triplet (T) and LLS state. T_T_ was found to be 7 ± 1 s whereas the lifetime for the pseudo singlet state contribution, T_LLS_, was 40 ± 3 s (r^2^ = 0.998). The individual proton T_1_ values for H2 and H3 were 28 and 18 s, respectively. The creation of a short‐lived singlet state is therefore indicated, with a signal lifetime of only ~1.7 times that of the average T_1_ because of the small couplings to proton sites H4 and H7.

It is noted that T_LLS_ values and T_1_ values were measured with and without the SABRE catalyst. The presence of the catalyst reduces signal lifetimes dramatically (Table 1). Therefore, removal of the catalyst would be required before use of LLS is further considered.^55^, ^56^

### 3.3 Optimization of SABRE polarization catalysis

**FIGURE 6:**
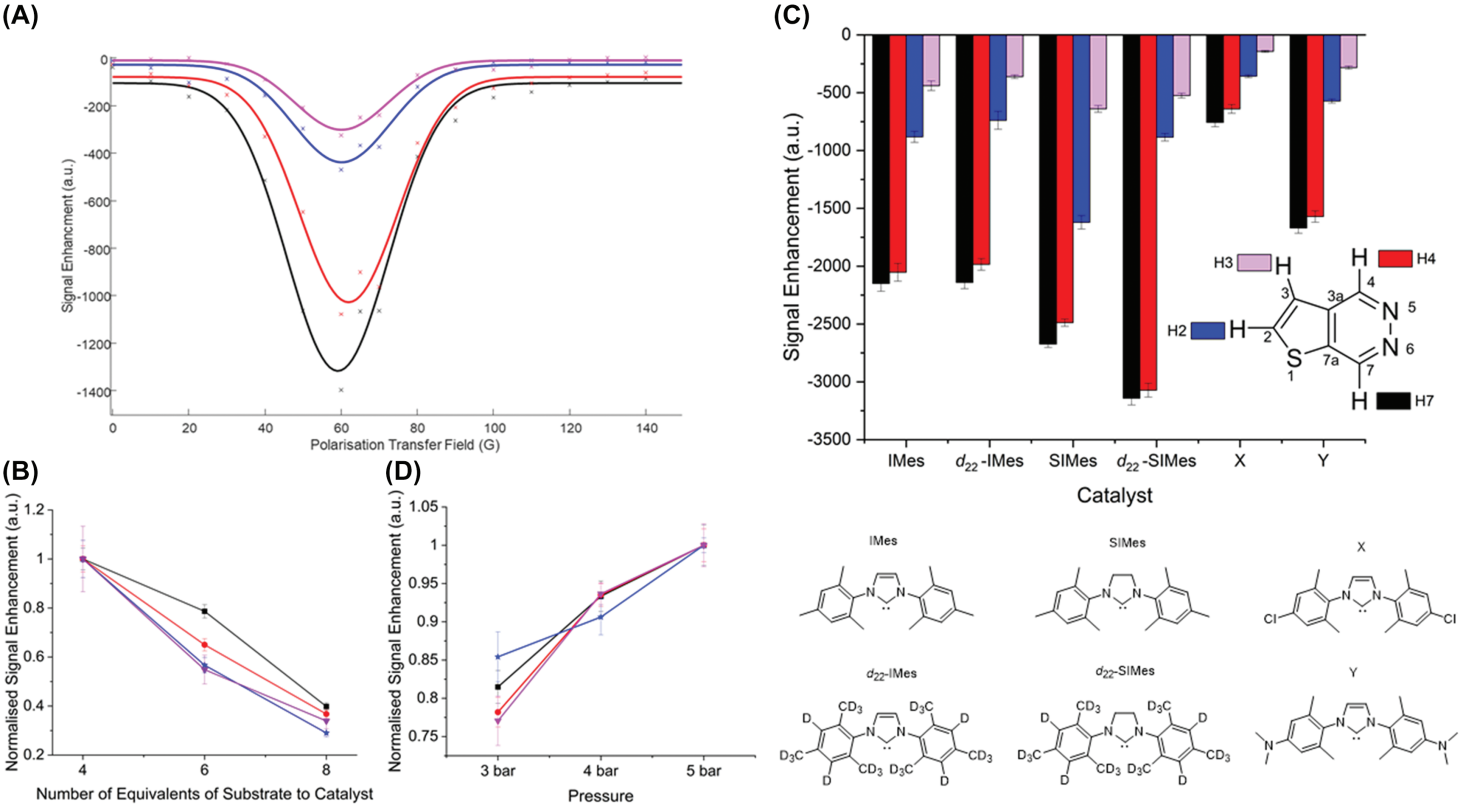
Optimization steps (A) polarization transfer field plot showing variation of signal enhancement for T[2,3‐*d*]P and [IrCl(COD)(IMes)] at 298 K using the automated polarizer. PTF plots for each resonance show a Gaussian distribution and give a single maximum at 60.4 ± 0.6 G. (B) Normalized enhancement values per individual proton with different substrate to catalyst [IrCl(COD)(IMes)] ratios for T[2,3‐*d*]P showing a linear decrease of enhancement with increased number of equivalents. (C) Graph showing the effect of a change of catalyst on the signal enhancement values of the four proton signals in T(2,3‐*d*)P with a 1:4 catalyst to substrate ratio. (D) Normalized signal enhancement factors of each of the four resonances (H7, H4, H2, and H3) of T[2,3‐*d*]P with [IrCl(COD)(*d*_22_‐SIMes)] at 3, 4, and 5 bar(g) of *p*‐H_2_ pressure. All measurements were made on a 400 MHz spectrometer at 298K

Previous studies on many hetero‐aromatic substrates have identified an optimum polarization transfer field (PTF) of around 65 G for ^1^H in the SABRE technique.^3^, ^49^, ^57^ This is reflective of the ^4^ *J*_HH_ couplings between hydride and ortho protons being 1.25 Hz in magnitude, the hydride‐hydride coupling of between 6 and 8 Hz and the chemical shift difference of around 28 ppm. A study of this field effect on the extent of hyperpolarization was completed for each of the four distinct protons of each substrate to identify this value more precisely. Resulting PTF data (Figure 6A) show that all substrates yield their largest signals between 60 G and 70 G. PTF plots for the higher symmetry substrates, T[2,3‐*d*]P and T[3,4‐*d*]P, are unimodal with a single maxima at 60.4 G ± 0.6 and 60.9 G ± 0.5, respectively, as predicted by the level anti‐crossing (LAC) approach.^43^, ^45^ PTF plots for all TPs can be found in the Supporting Information section 2.

It has been demonstrated that SABRE signal enhancement levels are affected by the molar ratio of the substrate to catalyst.^50^ To investigate the effect of substrate loading, samples were prepared using catalyst to substrate ratios 1:4, 1:6, and 1:8 for best performing T[2,3‐*d*]P. Figure 6B demonstrates that an optimum response was seen when four molar equivalents of substrate were present relative to the iridium precatalyst. This was also observed for T[3,4‐*d*]P (Supporting Information section 3).

Six catalysts precursors were then tested with T[2,3‐*d*]P. [IrCl(COD)(IMes)]^50^ and the saturated version of IMes, [IrCl(COD)(SIMes)],^50^ were used to investigate the effect of changing the steric bulk of the carbene ligand. The deuterated versions of [IrCl(COD)(*d*_22_‐IMes)]^51^ and [IrCl(COD)(*d*_22_‐SIMes)]^27^ were used to probe the effect of deuteration. [IrCl(COD)(X)]^27^ and [IrCl(COD)(Y)]^27^ were used to test electronic effects from the carbene. Replacement of [IrCl(COD)(IMes)] by [IrCl(COD)(*d*_22_‐SIMes)] ultimately resulted in the best performance with the signal enhancement levels of H7 within T[2,3‐*d*]P of −3142‐fold ± 58 (Figure 6C).

Because *p*‐H_2_ is consumed during catalysis, during a standard SABRE experiment carried out at 3 bar(g) of pressure, it becomes the limiting factor for enhancement as eventually all of it is converted to H_2_.^27^ To test this hypothesis, experiments were carried out with T[2,3‐*d*]P and [IrCl(COD)(*d*_22_‐SIMes)], changing only the pressure of the *p*‐H_2_ introduced into the standard 5 mm J. Young tube to 4 bar(g) and 5 bar(g) with a constant exposure (shake) time of 10 seconds. The results should, therefore, reflect the increase in *p*‐H_2_ incorporation rate into the SABRE catalyst. The results shown in Figure 6D support the theory that an increase in *p*‐H_2_ pressure leads to a corresponding increase in SABRE enhancement. An increase in signal enhancement with *p*‐H_2_ pressure is independent of the proton site (with all normalized values within standard error at each pressure value). An increase from 3 bar(g) to 5 bar(g) results in a 20% gain in signal enhancement across the substrate. It must be noted that the use of higher gas pressures is not practical for routine use without increasingly specialized apparatus. One can hypothesize that if equipment allowed an increase in the pressure beyond 5 bar(g) there would be a corresponding increase in enhancement before a plateau at a given pressure, at which point relaxation effects would dominate.

**FIGURE 7:**
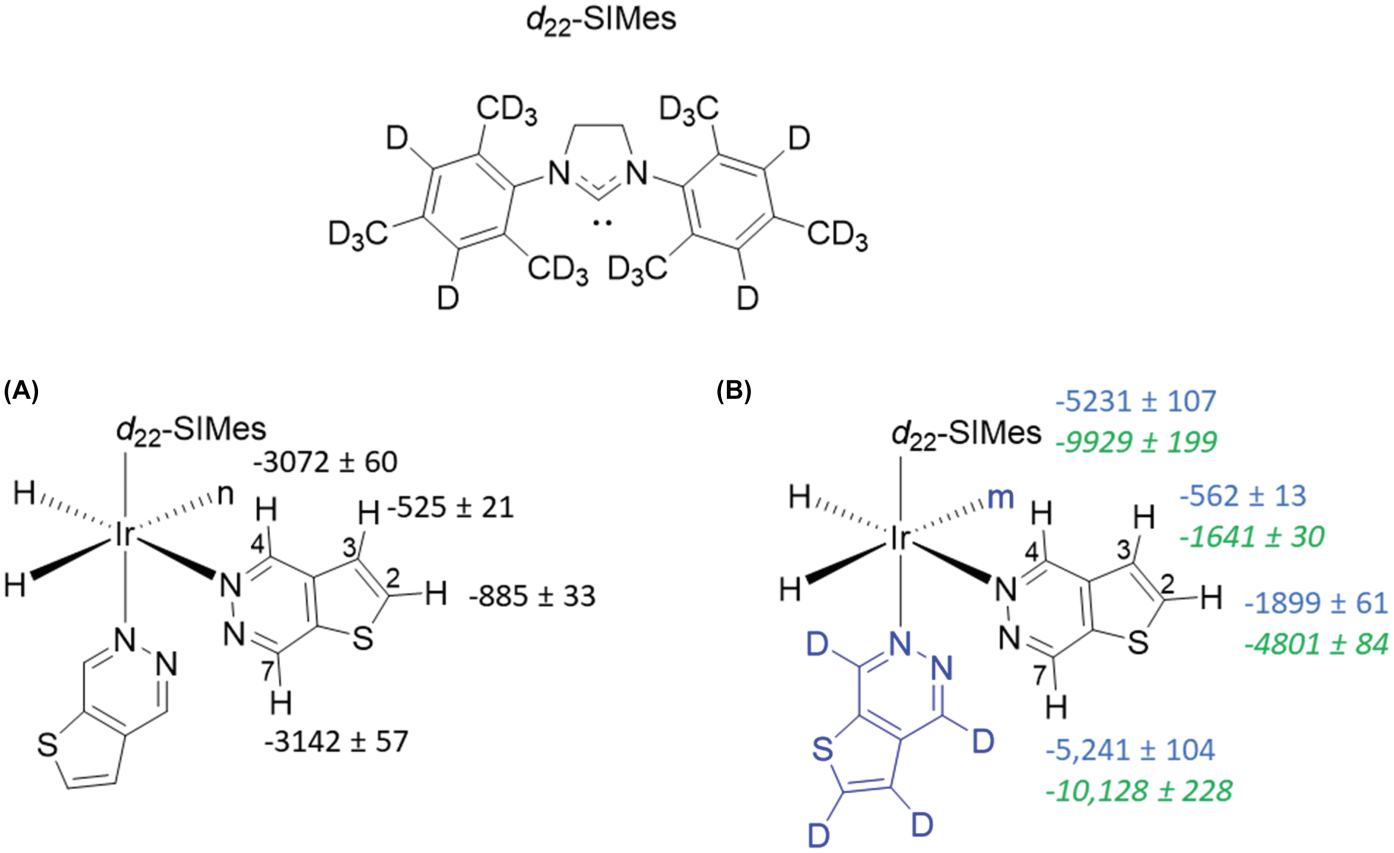
Comparison of hyperpolarization levels under (A) standard conditions of [IrCl(COD)(IMes)] and T[2,3‐*d*]P in a 1:4 catalyst : substrate ratio (B) using a co‐ligand with standard shake and drop (blue numbers) and using a co‐ligand and shaking with a 60 G handheld shaker (green numbers). When the co‐ligand was used it was in a 1:1:3 catalyst : substrate : deuterated co‐ligand with [IrCl(COD)(IMes)] as catalyst, T[2,3‐*d*]P as substrate and *d*_4_‐T[2,3‐*d*]P as deuterated co‐ligand. n = in plane T[2,3‐*d*]P, m = in plane *d*_4_‐T[2,3‐*d*]P (removed to aid the reader).

The presence of a fully deuterated co‐ligand (isotopologue of T[2,3‐*d*]P) resulted in a further increase in H7 signal enhancement to −5240‐fold at 9.4T (17% polarization), through reduction of spin dilution to these co‐ligands. Furthermore, when shaking in the somewhat variable stray field of the magnet was replaced by shaking in a purpose built 60 G handheld magnet array^46^ a further increase to −10,130 fold (33% polarization) resulted (Figure 7).

### 3.4 Imaging capability

**FIGURE 8:**
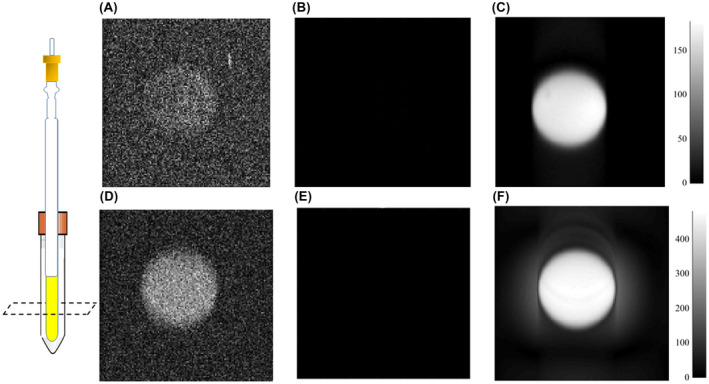
Thermally polarized RARE images of (A) 1:4 ratio of [IrCl(COD)(*d*_22_SIMes)] and T[2,3‐*d*]P in methanol‐*d*_4_, (D) 1:20 ratio; (B) and (E) are the same thermal images as (A) and (D) but normalized with respect to the hyperpolarized images (C) and (F). In the hyperpolarized images (C) and (F) 3 bar(g) of *p*‐H_2_ was used and the sample shaken for 10 s in a 60 G shaker before measurement at 9.4T

RARE imaging studies on optimally hyperpolarized T[2,3‐*d*]P were then completed on *in vitro* phantoms/models at 9.4T. Example images are shown in Figure 8. Figure 8A,D show thermal images for 1:4 and 1:20 sample ratios with S/N values of 1 ±1 and 3 ±1, respectively, (as expected 1:20 has more substrate and, therefore, higher signal). Figure 8C,F show the hyperpolarized images after shaking with 3 bar(g) of *p*‐H_2_ with S/N of 42 and 58 for the 1:4 and 1:20 ratio, respectively. Figure 8B,E show the thermal images with gray level normalized to the hyperpolarized scans. SABRE hyperpolarization is clearly observed in this *in vitro* model. Although the 1:20 sample ratio has the highest signal because of increased baseline level, actual signal gain is lower (19.41) compared to the 1:4 ratio (41.75). The blurring observed in both Figure 8C,F is caused by (1) the centric encoding scheme and (2) chemical shift artefacts caused by the four ^1^H resonances within the T[2,3‐*d*]P molecule.

#### 3.4.1 Magnetic resonance spectroscopy (CSI and EPSI)

**FIGURE 9:**
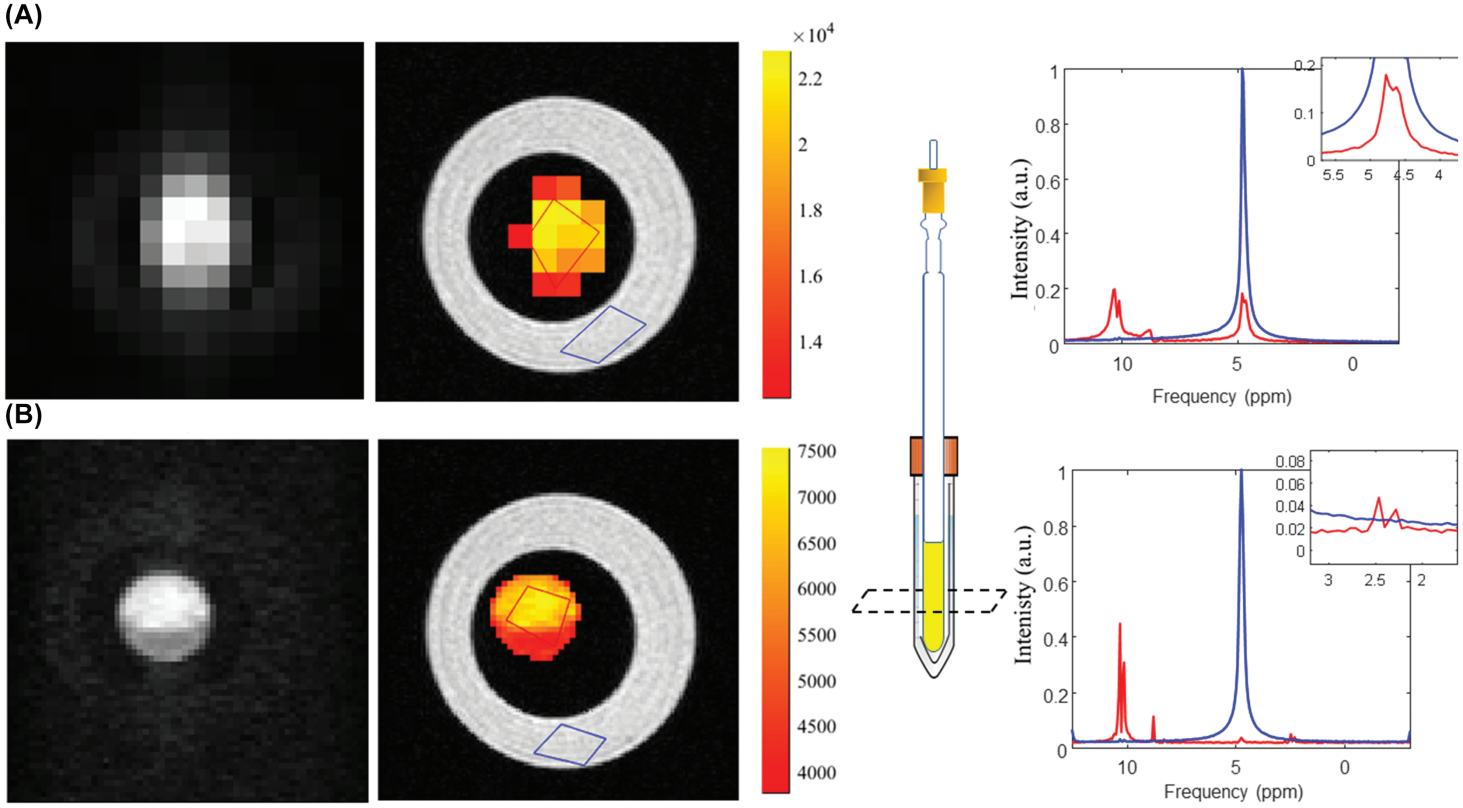
Comparison of (A) CSI (1 mm slice thickness) and (B) EPSI (1 mm slice thickness) showing image maps of hyperpolarized T[2,3‐*d*]P and spectra normalized to the water peak. Insert in CSI spectrum (A) shows the incurrence of the water peak within the hyperpolarized spectrum with similar amplitude to the peak of interest. Insert in EPSI spectrum (B) shows a Nyquist ghost of the T[2,3‐*d*]P peaks at δ 2.4

Although direct high‐resolution imaging of the proton resonances is permitted by ^1^H SABRE, it is recognized that for practical *in vivo* application the background proton signal from body water would confound such approaches or necessitate the use of selective saturation. Therefore, a spectroscopic approach may be preferred. To investigate the spectral imaging response and identify the multiple proton resonances within the T[2,3‐*d*]P molecule a chemical shift‐based sequence was used. A 20:1 T[2,3‐*d*]P sample was prepared with [IrCl(COD)(IMes)]. The J. Young’s tube was placed within a falcon tube containing water/agar to aid automated shimming and to mimic the water resonance that would be present in *in vivo* conditions. Subsequent spectra‐spatial data, captured using centric encoded CSI (74 s), is shown in Figure 9A.

A benefit of hyperpolarizing ^1^H sites is the relatively small bandwidth of possible resonances (~20 ppm). This permits the application of EPSI as a faster method of spectroscopic imaging for all resonances. A complementary EPSI experiment was also completed on the same sample. Total EPSI scan time was 6 s and the sequence parameters resulted in a 4‐fold increase in spatial resolution over the CSI equivalent (at a slight cost of spectral resolution and width, 18 Hz/pt across 15 ppm, compared with 15 Hz/pt across 30 ppm). A direct comparison between resulting CSI and EPSI acquisitions for detection of hyperpolarized T[2,3‐*d*]P was made (Figure 9A,B, respectively). The EPSI results show improved signal to noise (2× relative intensity to water) with less “leakage”/bleeding artefacts from the background water signal (see insert Figure 9A).

As shown in the spectrum of Figure 9B, a small 4% ghosting artefact is present at ~δ 2.4; demonstrating one of the disadvantages of the EPSI approach (setting correct gradient timings is difficult using time decaying signals).

## 4 DISCUSSION

### 4.1 ^1^H SABRE in NMR and optimization of SABRE polarization catalysis

Four thienopyridazines isomers were hyperpolarized using the SABRE technique. All isomers achieved high levels of SABRE hyperpolarization signal enhancements over thermal conditions. T[2,3‐*d*]P represented the optimum configuration for the greatest SABRE signal enhancement. The position of the nitrogen atoms relative to the five membered sulfur ring clearly plays a role in the efficiency of spin polarization transfer. For T[2,3‐*c*]P and T[3,2‐*c*]P one of the two possible nitrogen binding sites (position 7 in T[2,3‐*c*]P and position 4 in T[3,2‐*c*]P) is adjacent to the carbon of the fused rings and, therefore, sterically hindered when approaching the catalyst.

In agreement with previous studies, catalyst optimization remains substrate specific.^26^ For TP the use of a more flexible, sterically bulky carbene backbone (e.g., SIMes) gave the highest signal enhancements. Although it is unusual for [IrCl(COD)(SIMes)] to be the better mediator of polarization transfer compared with [IrCl(COD)(IMes)], there have been other rare examples where this is the case.^58^ It is noted that in all cases a significant amount of latent polarization remained in the bound substrate. With [IrCl(COD)(IMes)] and T[2,3‐*d*]P the highest signal enhancement −2150 ± 67 was measured for H7 on the free substrate. In the same spectrum, a bound peak was measured with an enhancement of −1576 ± 50 at 9.3 ppm. Moving this “bound” polarization into solution will maximize SABRE polarization levels. SIMes is known to have a faster rate of ligand exchange compared with its unsaturated analog.^50^ Therefore, a catalyst that provides an even greater rate of ligand exchange could result in greater polarization build‐up on the free substrate and hence less polarization being seen on its bound form. However, there is a balance as going to too great a dissociation rate may lower polarization levels.

Although it is difficult to establish ideal polarization conditions for specific substrates without completing optimization studies, some general trends are apparent. The relation between signal enhancement and *p*‐H_2_ pressure represents a simple route to increasing polarization.^44^ Although there are obvious practical safety considerations to using high pressure, any SABRE based polarization device for future clinical rollout should aim to safely work at pressures beyond 5 bar (g). Both experimental data^50^ and theoretical models predict that large signal enhancements result with low substrate‐to‐catalyst ratios.^44^ An increase in number of equivalents shows a corresponding decrease in signal enhancement independent of proton site. In the current study, all protons show over 50% reduction in signal enhancement when moving from 4 to 8 equivalents (see Supporting Information). However, this ratio means a relatively large amount of catalyst is required, which would require removal before delivery of the bolus in a clinical setting.

When considering SABRE translation to the clinic an automatic polarizer would be necessary to achieve consistent polarization levels. Polarization via an automated system in this study was lower than the standard manual shaking (NB: PTF data [Figure 6A] completed with the automated polarizer shows enhancement of −1300 vs. −2150 with manual shake for H7 of T[2,3‐*d*]P). This is driven by less efficient mixing with *p*H_2_, and exposure to the air as samples are shuttled to the detector. Further research is required to optimize this system to achieve at least the levels of polarization reached using the manual method. Data here support use of specifically designed magnetic shakers tuned to a specific polarization transfer field (~60 G) to further optimize polarization transfer.

Deuteration of both the catalyst and substrate are also clear routes to increased polarization (and longer T_1_ values). This is consistent with less spin dilution and reduced dipole‐dipole relaxation respectively because of the presence of deuterium. Here, use of a fully deuterated T[2,3‐*d*]P co‐ligand in a 1:3:1 catalyst to co‐ligand to substrate ratio afforded higher signal enhancements (66% increase). The co‐ligand does not receive polarization itself and, therefore, reduces spin dilution to give higher polarization levels on the ^1^H‐sites of the protio substrate trans to hydride in the active SABRE catalyst.

It is noted that although achieving 33% polarization for T[2,3‐*d*]P represents an impressive increase in potential sensitivity for *in vivo* detection, the reduced magnetic lifetime of the compound in the presence of the catalyst (9 s H7 at catalyst:substrate ratio of 1:4) is a limiting factor. Furthermore, these levels of polarization were achieved in a methanol‐*d*_4_ solvent, which is toxic and not suitable for subsequent *in vivo* injection (see Section 4.4).

### 4.2 Signal lifetime

A long‐lived singlet state pulse sequence was applied to the target thienopyridazines. The regioisomer (T[2,3‐*d*]P) showed an extension in magnetic lifetime in the proton pair of interest (between H2 and H3 from T_1_ of 18 and 28 s to T_LLS_ of 40 s). A 40‐s magnetic lifetime is in‐line with ^13^C d‐DNP polarized pyruvate and therefore supports clinical detection feasibility.^17^, ^59^ It was recognized that a potential pathway to further relaxation could exist if protons of the singlet state pair are scalar coupled to other protons within the same molecule. One way to overcome this would be to selectively deuterate H7 and H4. However, for T[2,3‐*d*]P, this could be counterproductive because H7 already has a T_1_ lifetime of 49 s (longer than the long‐lived lifetime on the H2/H3 resonances). In fact, deuterating H2, H3, and H4 in an effort to increase the T_1_ of H7 (without the use of LLS strategies) would be more sensible in this case. Unfortunately, synthetic attempts were unsuccessful.

Interestingly, elsewhere we tested 3,6‐substituted pyridazines, representing simpler systems (containing only one proton pair).^60^ Despite their limited SABRE hyperpolarization (under the transfer conditions used)^60^ neighboring ^1^H nuclei in three pyridazine molecules tested were extended by up to 3× their individual T_1_ lifetimes. 3‐Chloro‐6‐methoxypyridazine (with inherent T_1_ values of 31 and 29 seconds) yielded a T_LLS_ value of over 100 s highlighting how effective LLS can be.

### 4.3 ^1^H SABRE and imaging

High hyperpolarized signal intensities were observed with a single shot ^1^H RARE imaging approach. One of the advantages of hyperpolarizing protons is that one can use the abundant water signal on a phantom to easily optimize the power settings for the transmit coil using standard approaches. This means setting the accurate pulse power for a π pulse is possible and rapid spin echo measurements can be completed. Unfortunately, RARE approaches do not easily encode chemical shift information and resultant images exhibit chemical shift artefacts.

To overcome chemical shift artefacts, MRSI can be used. Both CSI and EPSI techniques allow chemical shift information to be mapped spatially. It is noted that using a more spectroscopic imaging approach, hyperpolarized signals of up to 40% of the background ^1^H water signal were detected (even when said background protons were saturated with the fast RF pulsing regimes applied to overcome hyperpolarization T_1_ decay). Volumes would need to be scaled up for detection in humans and might affect polarization levels.

In both cases, RF suppression of the water pool could be used to ease the detection of the hyperpolarized signal. Such approaches are standard in thermal based MRS, but successful implementation takes time, requires a localized voxel shimming approach, and uses high flip angle pulses.^61^, ^62^, ^63^, ^64^, ^65^ High flip angle RF pulses, when applied during a SABRE based experiment could potentially attenuate the desired hyperpolarization signal (especially *in vivo* where field shimming is compromised) and therefore would be somewhat detrimental.

When considering hyperpolarized MR imaging, overall scan time and image resolution are very important considerations. CSI, as applied here, has a long >1‐minute scan time, with low in plane spatial resolution (16 × 16 pixels, ~2.2 mm^2^). Long scan times are a problem when considering a hyperpolarized agent with a relatively short T_1_. To overcome this limitation centric (fast radial/spiral) encoding schemes should always be used.^66^, ^67^ EPSI has a relatively short scan time in comparison (~6 s) and better spatial resolution (~0.5 mm^2^), albeit at the slight expense of spectral resolution and potential N/2 Nyquist ghosting. The reduction in spectral resolution is not a major problem for ^1^H MRS as the spectral width is narrow (20 ppm). This would not be true for X nuclei, such as ^13^C, where the spectral width required for metabolic mapping is much wider (~200 ppm).^6^ Nyquist ghosting occurred in the EPSI results presented here because of incorrect parametrization of the gradient delay times. Analyzing the odd and even echoes separately to correct for the N/2 ghost artefacts was possible, but resulted in half the sweep width.^68^ The water peak will alias into the detection window and confound the T[2,3‐*d*]P peak identification. This can be manually corrected when the peaks arise in spatially distinct areas, but *in vivo* this would not be the case and more sophisticated correction methods would have to be implemented.^69^, ^70^ It is noted that these concept imaging studies did not use a fully deuterated co‐ligand (which delivers the highest polarization levels). Synthesis of the fully deuterated ligand was only completed on a small scale. A cost benefit analysis would be required to use deuterated molecules large‐scale.

### 4.4 Biocompatibility

Although SABRE has proven to deliver high levels of polarization^27^, ^71^ across a range of molecules, often this is within organic solvents (for increased dissolution of both *p*‐H_2_ and the Ir based catalyst) not suitable for biomedical use. Although methods to address this should be the primary focus of future research, it is noted that the deuteration of the actual ligand/drug (to enhance target polarization) could alter biological activity and should be investigated.

Direct SABRE polarization in water would be ideal, however both the metal catalyst [IrCl(COD)(IMes)] and *p*‐H_2_ ^72^ have poor solubility. To reduce toxicity, previous studies have used solvent mixtures of ethanol‐*d*_6_ and D_2_O.^73^ However, the level of signal gain is reduced^49^ and T_1_ relaxation values were found to decrease relative to those in methanol.^27^ The lower enhancement values reflect the change in ligand exchange kinetics.^49^ Toxicity studies show a maximum of 30% ethanol can be tolerated before cell death occurs.^55^ Biphasic separation permits isolation of the hyperpolarized agent in an aqueous phase while the toxic iridium catalyst remains in the organic phase. The separation of the catalyst from the hyperpolarized substrate also means T_1_ values will extend as they return to their original values. Preliminary experiments using the catalyst‐separated CASH‐SABRE^74^ method were carried out on T[2,3‐*d*]P using a D_2_O saline bolus. A hyperpolarized signal was observed in the aqueous layer, albeit under acidic conditions (pH 1.5), with a −940‐fold signal enhancement (3% polarization for H7) compared to the corresponding methanol‐*d*_4_ value of make −3142‐fold (9.8% polarization). Hence, the biphasic route appears to be effective for delivering biocompatible SABRE, but alternatives using water‐soluble catalysts,^75^, ^76^, ^77^, ^78^ phase extraction with scavenging agents^79^ or chelating ligands may need to be considered.^55^

The present study also uses deuterated TP motifs as co‐ligands to concentrate spin hyperpolarization on the protio version. One would expect similar deuteration of the potential drug scaffolds may be required for this reason. Selective deuteration (specifically formation of strong C‐^2^H bonds) may lead to known kinetic isotope effects^80^, ^81^ and altered toxicity. Deuterated molecule interactions with enzymes often lead to very different pharmo‐kinetics, compared to non‐deuterated standards.^82^, ^83^ However, this phenomenon can be exploited, resulting in deuterated drugs that retain their therapeutic effectiveness for much longer and consequently require lower or less frequent dosage. Indeed, deutetrabenazine is US Food and Drug Administration‐approved, and the altered metabolic pathways of the deuterated drug leads to reduced toxicity.^84^ Although the literature concerning the effects of drug deuteration remains conflicted^85^, ^86^, ^87^, ^88^ we have previously shown *in vivo* that the pharmo‐kinetic parameters of 4,6‐d_2_‐nicotinamide (one of the earlier SABRE hyperpolarization targets) are similar to protio‐nicotinamide. Because this target substrate was tolerated and deemed safe for administration,^89^ one could hypothesize similar findings for TP. This would have to be confirmed with more in‐depth toxicology screening.

### 4.5 Metabolism and subsequent detection

Once biocompatibility is achieved, initial chemical shift resolved NMR studies investigating hepatic metabolism *in vivo* will need to be completed. Our search of the literature found no specific data on the hepatic metabolism of TP scaffolds. However, this structure is comparable to that of thienopyridine, the metabolism of which is well‐documented.^90^ It would, therefore, be reasonable to hypothesize similar oxidative metabolism for TP. The resulting 2‐oxy form of the thiophene ring or the formation of N‐oxides would be expected to induce significant chemical shift changes, making ^1^H NMR sensible as a probe for these metabolic markers. This would need to be tested. If ^1^H is not sufficiently sensitive (because of abundant background signals/subtle shift differences) one could target ^15^N instead. d‐DNP hyperpolarization studies already take advantage of its broader chemical shift range, and longer T_1_ lifetimes.^91^ It is noted that synthesis of a ^15^N labeled version of T[2,3‐*d*]P is possible.

Chemically active substituent groups attached to the TP backbone (to form a suitable drug), or direct TP binding to target enzymes (e.g., IKK or CHK1/CHK2), could shorten T_1_ lifetimes. We hypothesize that such alterations in chemical structure and/or conformation would result in chemical shift changes of similar magnitude to that observed when the TP ligand binds to the SABRE catalyst (Figure 4). Mandal et al^92^ have used ^1^H SABRE hyperpolarization to explore protein‐ligand interactions.^92^ Notwithstanding, in the absence of significant metabolism, one could also envisage using the expected physical changes in T_1_ relaxation time to potentially track important kinetics (i.e., a bi‐exponential signal decay model account for bound and free TP).

## 5 CONCLUSION

In summary, a family of thienopyridazines, with known anticancer properties, have been successfully studied with ^1^H SABRE hyperpolarization of 33% (H7) recorded for T[2,3‐*d*]P. Unlike alternative hyperpolarization methods, SABRE provides a simple, rapid, reversible, and inexpensive route, capable of polarizing a wide range of molecules containing NMR active nuclei (^1^H, ^13^C, ^15^N, and ^31^P). Targeting ^1^H as the probe nucleus (as opposed to ^13^C or ^15^N) is beneficial because it (1) has the highest gyromagnetic ratio of all nuclei and, therefore, high NMR sensitivity; (2) has a 99.98% natural abundance (negating the need for expensive/complicated synthetic pathways to generate the agents); and (3) can be used with existing clinical ^1^H MRI systems without the need for expensive hetero‐nuclei RF coils/system upgrades. We have shown how this low‐cost hyperpolarization technique can be combined with MRS techniques available on all standard MRI scanners. These early‐stage developments form a solid springboard for continued research and eventual clinical application of this technology for hyperpolarized drug tracking/chemotherapy.

## Supporting information


**FIGURE S1** Structure of *d*_4_‐thieno[2,3‐*d*]pyridazine
**FIGURE S2** Polarization transfer field plot for T[3,4‐*d*]P at 298 k using the automated polarizer. PTF plots for each resonance show a Gaussian distribution and give a single maximum at 60.9 ± 0.5 G
**FIGURE S3** Polarization transfer field plot for T[2,3‐*c*]P at 298 k using the automated polarizer. PTF plots for each resonance show a Gaussian fit and give three maxima at peak 1 = 13.30 ± 4.48 G, peak 2 = 65.37 ± 0.60 G, and peak 3 = 109.778± 0.13 G
**FIGURE S4** Polarization transfer field plot for T[3,2‐*c*]P at 298 K using the automated polarizer. PTF plots for each resonance show a Gaussian fit and give three maxima at peak 1 = 13.64 ± 1.06, peak 2 = 60.81 ± 0.62, and peak 3 = 109.36 ± 0.81983
**FIGURE S5** Hyperpolarized spectra for T[3,2‐*c*]P achieved at 0G (blue) and 60 G (black). Positive magnitudes for H7 and H3 and negative magnitudes for H6 and H2 at 0 G (blue). Negative magnitudes for all resonances at 60 G (black)
**FIGURE S6** Effect on signal enhancement values when changing substrate to catalyst ratios. The substrate used was T[3,4‐*d*]P and the catalyst [IrCl(COD)(IMes)] forming the active catalyst [Ir(H)_2_(IMes)(T[3,4‐*d*]P)_3_]Cl. The solvent used was methanol‐*d*_4_ and measurements were made on a 400 MHz spectrometer at 298K. (A) Signal enhancements values measured per individual proton with 4, 6, and 8 eq. of T[3,4‐*d*]P to 1 equivalent of catalyst [IrCl(COD)(IMes)]. (B) Normalized signal enhancement values per proton showing a linear decrease of enhancement with increased number of substrate equivalents
