# Supplementary material for: SABRE hyperpolarized anticancer agents for use in 1H MRI
Source: Magn Reson Med. 2022 Mar 7;88(1):11–27. doi: 10.1002/mrm.29166 (PMC9310590; doi:10.1002/mrm.29166)
Supplement: Supplementary file 1 — FIGURE S1 Structure of d 4‐thieno[2,3‐d]pyridazine FIGURE S2 Polarization transfer field plot for T[3,4‐d]P at 298 k using the automated polarizer. PTF plots for each resonance show a Gaussian distribution and give a single maximum at 60.9 ± 0.5 G FIGURE S3 Polarization transfer field plot for T[2,3‐c]P at 298 k using the automated polarizer. PTF plots for each resonance show a Gaussian fit and give three maxima at peak 1 = 13.30 ± 4.48 G, peak 2 = 65.37 ± 0.60 G, and peak 3 = 109.778± 0.13 G FIGURE S4 Polarization transfer field plot for T[3,2‐c]P at 298 K using the automated polarizer. PTF plots for each resonance show a Gaussian fit and give three maxima at peak 1 = 13.64 ± 1.06, peak 2 = 60.81 ± 0.62, and peak 3 = 109.36 ± 0.81983 FIGURE S5 Hyperpolarized spectra for T[3,2‐c]P achieved at 0G (blue) and 60 G (black). Positive magnitudes for H7 and H3 and negative magnitudes for H6 and H2 at 0 G (blue). Negative magnitudes for all resonances at 60 G (black) FIGURE S6 Effect on signal enhancement values when changing substrate to catalyst ratios. The substrate used was T[3,4‐d]P and the catalyst [IrCl(COD)(IMes)] forming the active catalyst [Ir(H)2(IMes)(T[3,4‐d]P)3]Cl. The solvent used was methanol‐d 4 and measurements were made on a 400 MHz spectrometer at 298K. (A) Signal enhancements values measured per individual proton with 4, 6, and 8 eq. of T[3,4‐d]P to 1 equivalent of catalyst [IrCl(COD)(IMes)]. (B) Normalized signal enhancement values per proton showing a linear decrease of enhancement with increased number of substrate equivalents [file MRM-88-11-s001.docx]

# Supporting Information

# Synthesis of *d*_4_-thieno[2,3-*d*]pyridazine


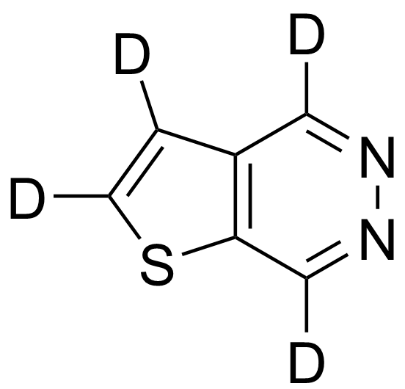


Supporting Information Figure 1: Structure of *d*_4_-thieno[2,3-*d*]pyridazine

Thieno[2,3-d]pyridazine (100 mg, 0.73 mmol ) was added to D_2_O (10 mL) with K_2_CO_3_ (245 mg, 1.7 mmol) and refluxed for 4 weeks. During this time, the deuteration reaction was followed by LC-MS. When there was little further conversion, the D_2_O was removed under reduced pressure and replenished with fresh D_2_O (15 mL x 6) and additional K_2_CO_3_. After cooling to room temperature, CH_2_Cl_2_ (20 mL) was added. The organic layer was separated, and the aqueous layer extracted with CH_2_Cl_2_ (2 x 20 mL). The combined organic phases were dried (MgSO_4_) and concentrated under reduced pressure to give *d*_4_-thieno[2,3-*d*]pyridazine (77.8 mg, 0.55 mmol, 75% with 94% deuteration) as a white solid; **IR** (ATR, cm^-1^) ν_max_ 2693w, 2917w, 2849w, 2317w, 2274m, 1422m, 1241s, 1018s, 797s 609m;  **^13^C NMR** (126 MHz, CD_3_OD, 298 K) δ 146.4 (t, *J* = 28.36 Hz), 146.3 (t, *J* = 28.36 Hz), 139.9 (s), 136.9 (s), 135.2 (t, *J* = 26.20 Hz), 121.9 (t, *J* = 27.2 Hz); **MS** (ESI) *m/z* 141 [(M+H)^+^_,_ 100], ; **HRMS** (ESI) *m/z* [M+H]^+^ calculated for C_6_HD_4_N_2_S 141.0419, found 141.0416 (3 ppm Mean error) *m/z* [M+H]^+^ calculated for C_6_D_4_N_2_NaS 163.0238, found 163.0236 (3 ppm Mean error). Synthesis of this product is novel and no existing literature was found.

23% is 140 C_6_H_2_D_3_N_2_S – divided across 4 proton sites therefore 94 % deuteration

# PTF plots for T[3,4-d], T[2,3-c], T[3,2-c] with [IrCl(COD)(IMes)]


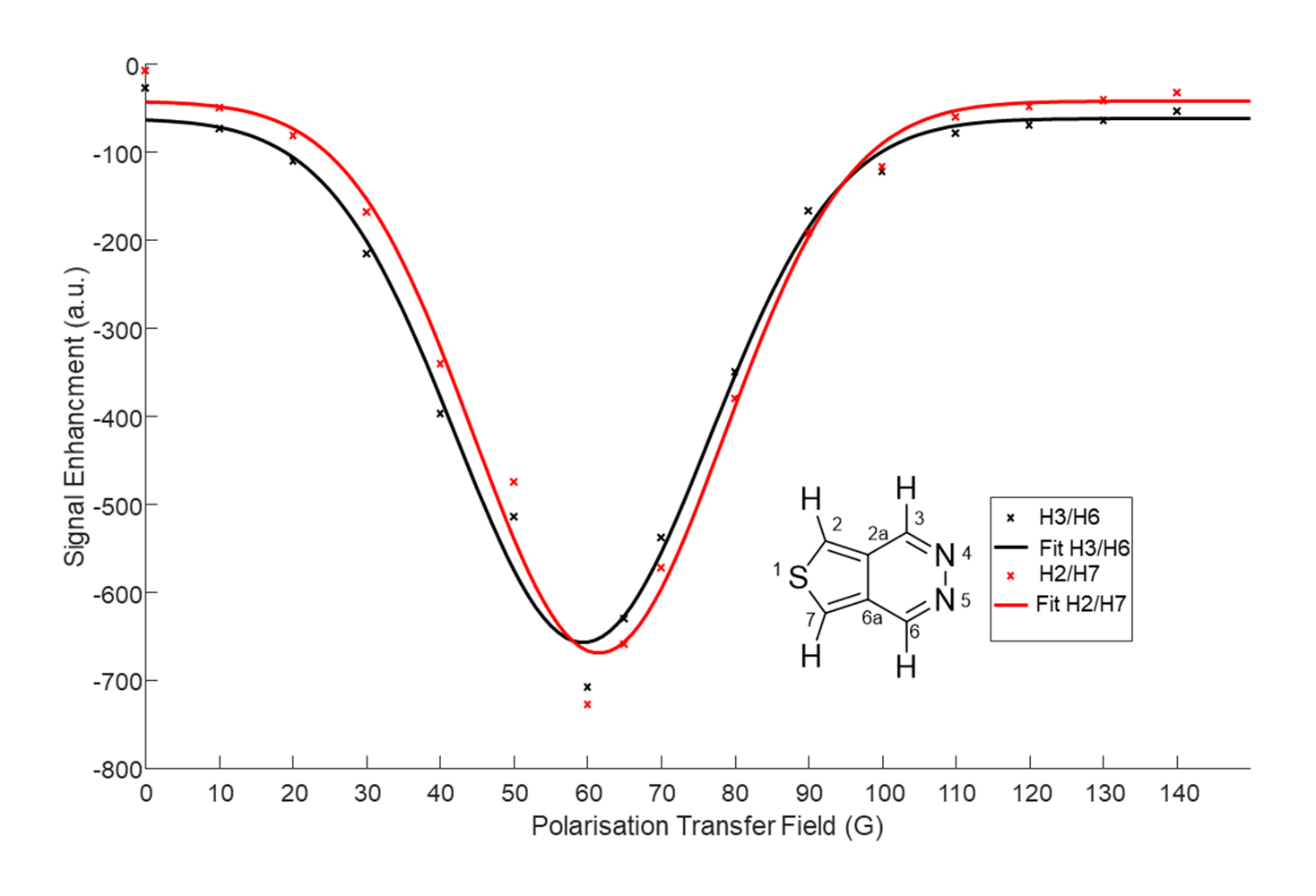


Supporting Information Figure 2: Polarisation transfer field plot for T[3,4-*d*]P at 298 k using the automated polariser. PTF plots for each resonance show a Gaussian distribution and give a single maximum at 60.9 +/- 0.5 G

When T[2,3-*c*] and T[3,2-*c*] were examined (Supplementary Figure 3 and Supplementary Figure 4) multimodal behaviour across polarisation field was observed. Three Gaussian functions were required to adequately fit the resulting PTF profiles. This is inconsistent with the normal LAC suggestions.^1^ Polarisation transfer was observed at lower fields; with positive amplitude for H7 and H3 and negative amplitude for H6 and H2 (Supplementary Figure 5). This trend has been modelled in the LAC theory, however a phosphorous ligand was present in the SABRE catalyst.^1^


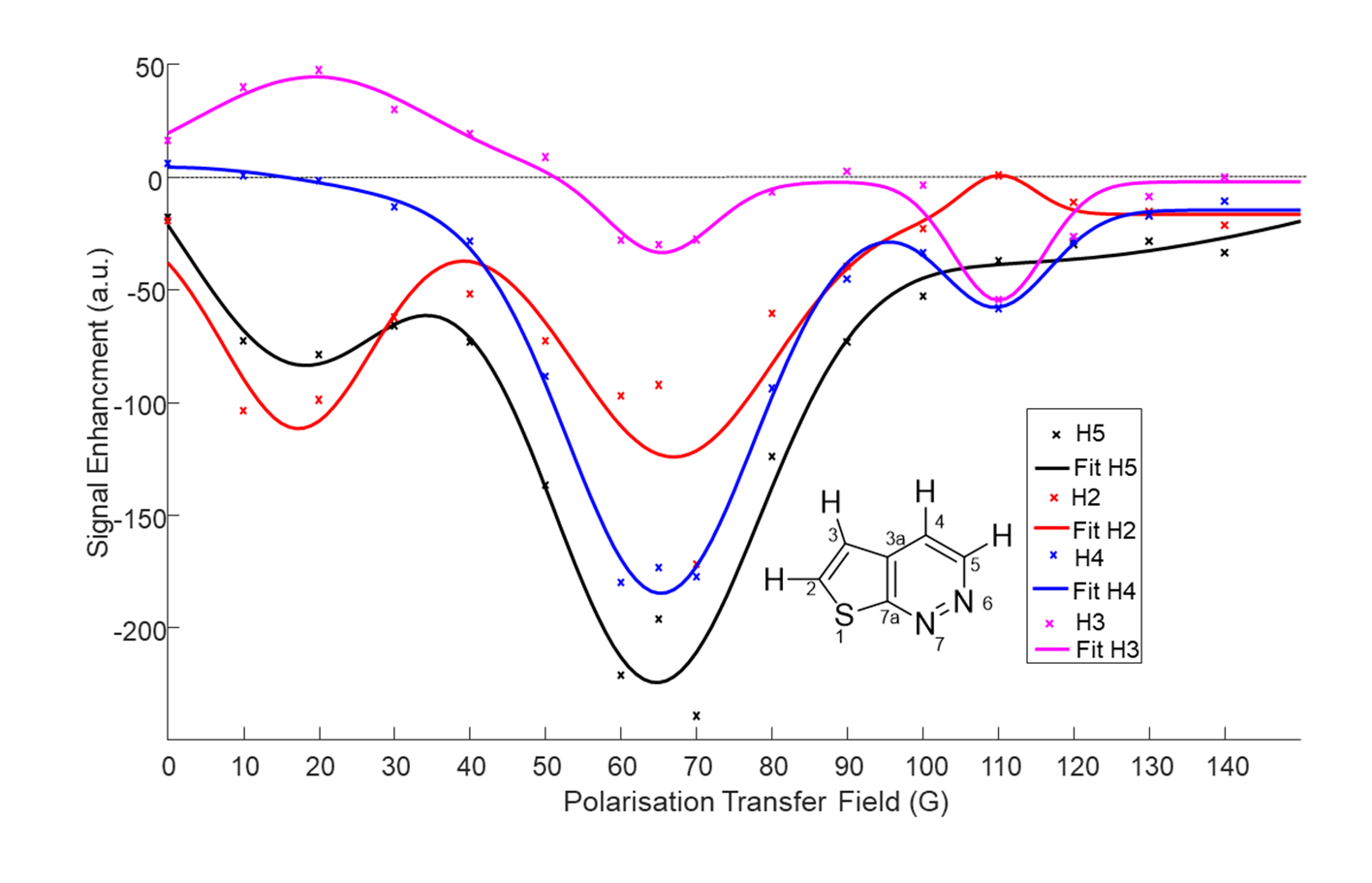


Supporting Information Figure 3: Polarisation transfer field plot for T[2,3-*c*]P at 298 k using the automated polariser. PTF plots for each resonance show a Gaussian fit and give three maxima at peak 1 = 13.30 +/- 4.48 G, peak 2 = 65.37 +/- 0.60 G and peak 3 = 109.778+/- 0.13 G


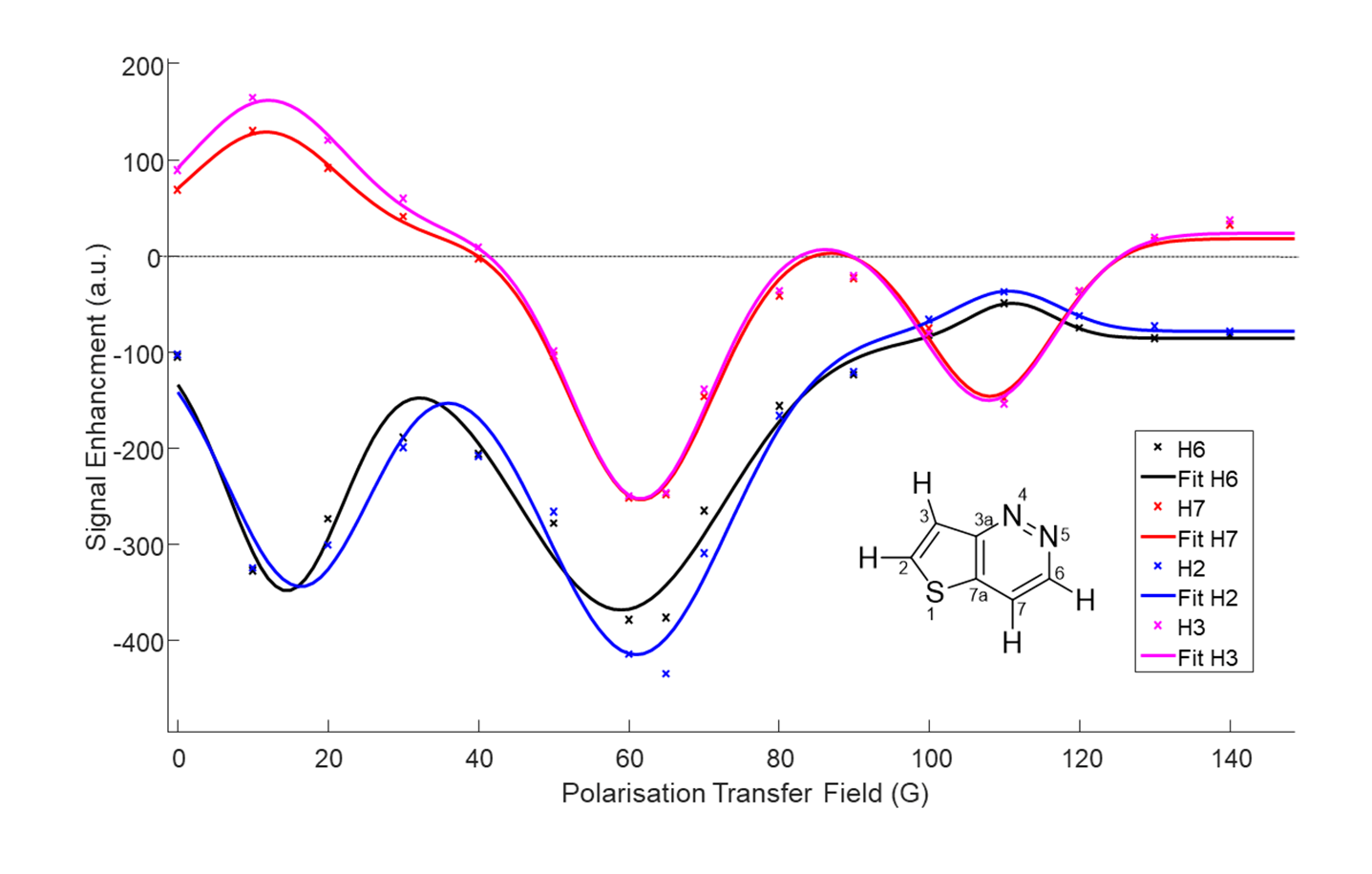


Supporting Information Figure 4: Polarisation transfer field plot for T[3,2-*c*]P at 298 K using the automated polariser. PTF plots for each resonance show a Gaussian fit and give three maxima at peak 1 = 13.64 +/- 1.06, peak 2 = 60.81 +/- 0.62 and peak 3 = 109.36 +/- 0.81983


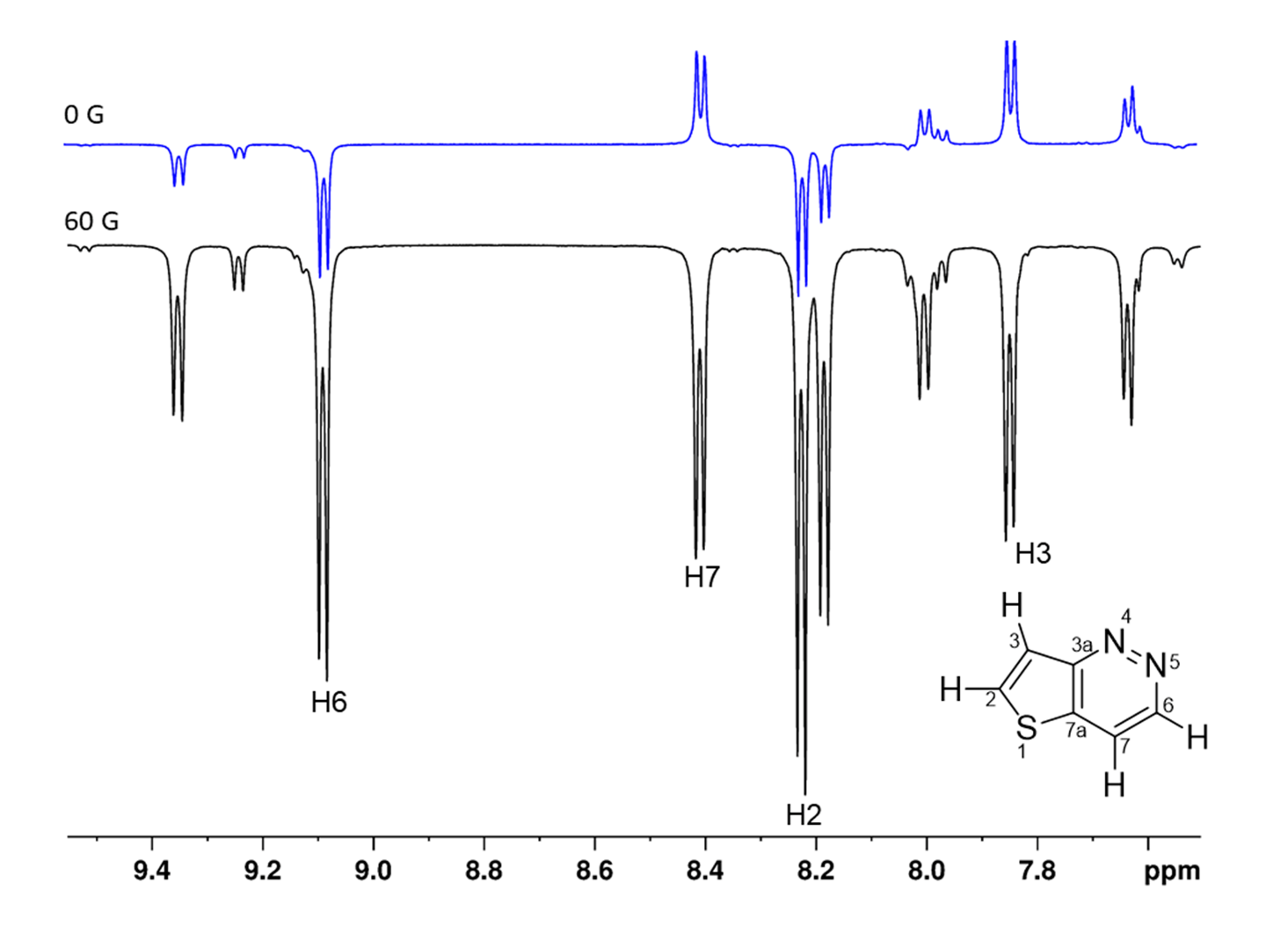


Supporting Information Figure 5: Hyperpolarised spectra for T[3,2-*c*]P achieved at 0G (blue) and 60 G (black). Positive magnitudes for H7 and H3 and negative magnitudes for H6 and H2 at 0 G (blue). Negative magnitudes for all resonances at 60 G (black)

The hydride region of the NMR spectra of all these samples is complex and consistent with several different SABRE active catalysts. One hypothesis to explain this behaviour is associated with the change in sign of the propagating *J*_HH_ couplings from the hydride to the protons in the ligand according to which of the two nitrogen binding sites is involved.^2-5^ This sign change is brought about by the resulting variation of coupling bond-order and will, in turn, invert the sign of the population difference, thereby leading to a hyperpolarised signal of opposite phase. It has been recently confirmed experimentally and theoretically that the changes in coupling constants resulting from binding to iridium lead to complex PTF profiles when mixtures of species are present in solution.^6^ Consequently, the resulting PTF profile should exhibit complex behaviour as contributions from the different forms of the catalyst combine together here to yield the resulting catalyst-averaged signal. This behaviour would require further very detailed theoretical and experimental study if a rigorous model were to be proposed.

# Substrate Loading Comparisons


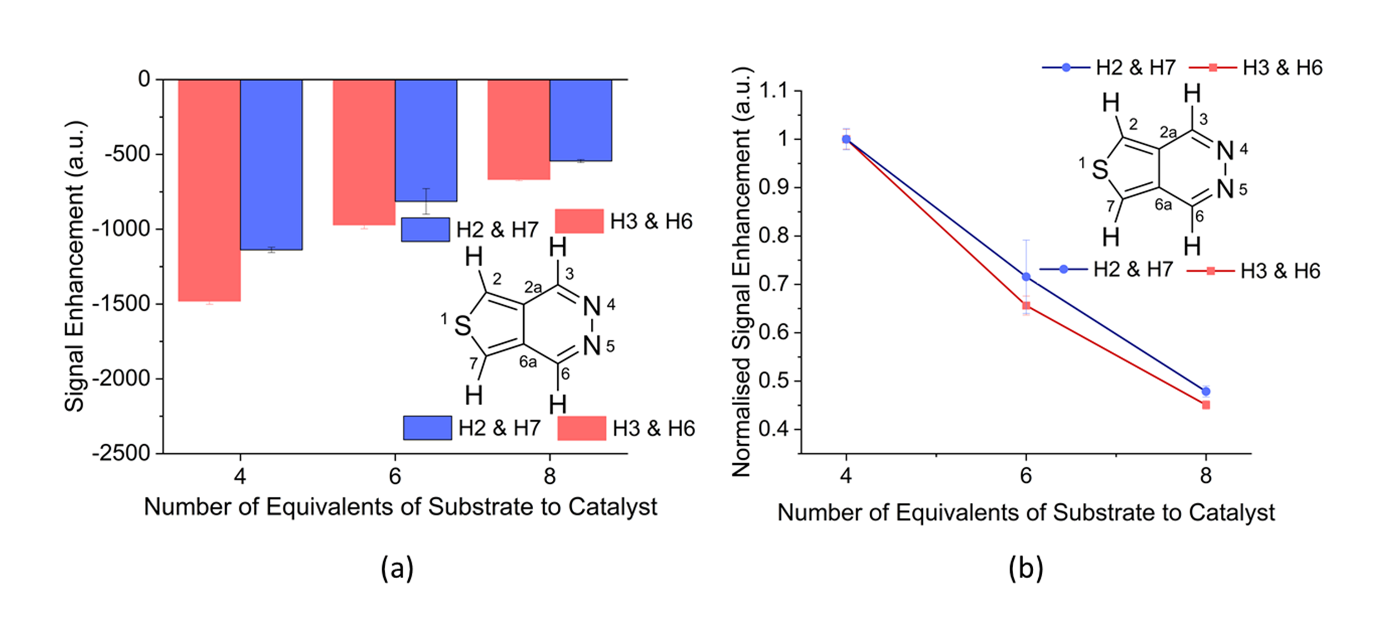


Supporting Information Figure 6: Effect on signal enhancement values when changing substrate to catalyst ratios. The substrate used was T[3,4-*d*]P and the catalyst [IrCl(COD)(IMes)] forming the active catalyst [Ir(H)_2_(IMes)(T[3,4-*d*]P)_3_]Cl. The solvent used was methanol-*d*_4_ and measurements were made on a 400 MHz spectrometer at 298K. (a) Signal enhancements values measured per individual proton with 4, 6 and 8 eq. of T[3,4-*d*]P to 1 equivalent of catalyst [IrCl(COD)(IMes)]. (b) Normalised signal enhancement values per proton showing a linear decrease of enhancement with increased number of substrate equivalents.

The linear dependence of equivalence was also observed for all proton sites within T[3,4-*d*]P evidenced by the normalised data in Supplementary Figure 6b. The resonance for the equivalent protons again shows 50% reduction in signal enhancement when moving from four to eight equivalents of substrate to catalyst.

1. Pravdivtsev AN, Yurkovskaya AV, Vieth HM, Ivanov KL, Kaptein R. Level Anti-Crossings are a Key Factor for Understanding para-Hydrogen-Induced Hyperpolarization in SABRE Experiments. *Chemphyschem*. Oct 2013;14(14):3327-3331. doi:10.1002/cphc.201300595

2. Ivanov KL, Pravdivtsev AN, Yurkovskaya AV, Vieth H-M, Kaptein R. The role of level anti-crossings in nuclear spin hyperpolarization. *Progress in Nuclear Magnetic Resonance Spectroscopy*. Aug 2014;81:1-36. doi:10.1016/j.pnmrs.2014.06.001

3. Barskiy DA, Knecht S, Yurkovskaya AV, Ivanov KL. SABRE: Chemical kinetics and spin dynamics of the formation of hyperpolarization. *Progress in nuclear magnetic resonance spectroscopy*. 2019 Oct - 2019;114-115:33-70. doi:10.1016/j.pnmrs.2019.05.005

4. Ducker EB, Kuhn LT, Munnemann K, Griesinger C. Similarity of SABRE field dependence in chemically different substrates. *J Magn Reson*. Jan 2012;214:159-165. doi:10.1016/j.jmr.2011.11.001

5. Eshuis N, Aspers R, van Weerdenburg BJA, et al. Determination of long-range scalar H-1-H-1 coupling constants responsible for polarization transfer in SABRE. *Journal of Magnetic Resonance*. Apr 2016;265:59-66. doi:10.1016/j.jmr.2016.01.012

6. Fekete M, Roy SS, Duckett SB. A role for low concentration reaction intermediates in the signal amplification by reversible exchange process revealed by theory and experiment. *Physical Chemistry Chemical Physics*. Mar 2020;22(9):5033-5037. doi:10.1039/c9cp06386b
